# Supplementary material for: End Sequence Analysis Toolkit (ESAT) expands the extractable information from single-cell RNA-seq data
Source: Genome Res. 2016 Oct;26(10):1397–410. doi: 10.1101/gr.207902.116 (PMC5052061; doi:10.1101/gr.207902.116)
Supplement: Supplemental Material [file supp_gr.207902.116_Supp_Materials.tar.gz › ESAT-esat_v0.1/doc/html/classbroad_1_1core_1_1math_1_1_statistics-members.html]

ESAT: Member List


ESAT


- Main Page
- Related Pages
- Packages
- Classes
- Files

- Class List
- Class Index
- Class Hierarchy
- Class Members

All Classes Namespaces Files Functions Variables Enumerator Pages

- broad
- core
- math
- Statistics

broad.core.math.Statistics Member List

This is the complete list of members for broad.core.math.Statistics, including all inherited members.

|  |  |  |
| --- | --- | --- |
| absFold(Collection< Double > g1, Collection< Double > g2, boolean log) | broad.core.math.Statistics | static |
| absFold(double[] g1, double[] g2, boolean log) | broad.core.math.Statistics | static |
| absoluteValue(Matrix permutations) | broad.core.math.Statistics | static |
| absoluteValue(double[] observed) | broad.core.math.Statistics | static |
| anovaFStat(Collection< double[]> lists) | broad.core.math.Statistics | static |
| average(Collection<?extends Number > values) | broad.core.math.Statistics | static |
| average(double[] values) | broad.core.math.Statistics | static |
| computePermutedZScores(double observation, double[] controlObservations, double fudge\_factor) | broad.core.math.Statistics | static |
| computePermutedZScores(double observation, double[] controlObservations) | broad.core.math.Statistics | static |
| covariance(List<?extends Number > values1, List<?extends Number > values2, double mean1, double mean2) | broad.core.math.Statistics | static |
| covariance(List<?extends Number > values1, List<?extends Number > values2) | broad.core.math.Statistics | static |
| diff(double[] g1, double[] g2) | broad.core.math.Statistics | static |
| entropy(double[] p\_vec) | broad.core.math.Statistics | static |
| euclideanDistance(double[] x, double[] y) | broad.core.math.Statistics | static |
| euclideanDistance(List< Double > x, List< Double > y) | broad.core.math.Statistics | static |
| FDR(EmpiricalDistribution observedDist, EmpiricalDistribution[] randomDist, double x) | broad.core.math.Statistics | static |
| FDRCorrect(double[] pvals, double alpha) | broad.core.math.Statistics | static |
| FDRCorrect(List< Double > pvals, double alpha) | broad.core.math.Statistics | static |
| fold(Collection< Double > g1, Collection< Double > g2, boolean log) | broad.core.math.Statistics | static |
| fold(double[] g1, double[] g2, boolean log) | broad.core.math.Statistics | static |
| fold(double[] g1, boolean log) | broad.core.math.Statistics | static |
| geometricMean(double[] vals) | broad.core.math.Statistics | static |
| geometricMeanMAD(Matrix data) | broad.core.math.Statistics | static |
| getKSStatistic(double[] observed, double[] expected) | broad.core.math.Statistics | static |
| hamming(double[] x, double[] y, double threshold) | broad.core.math.Statistics | static |
| hamming(List< Double > x, List< Double > y, double threshold) | broad.core.math.Statistics | static |
| hochbergPvalueAdjust(double[] pvals) | broad.core.math.Statistics | static |
| hypergeometric(int numberOfGenesTotal, int diffExpressedGenes, int genesInSet, int diffExpressedGenesInSet) | broad.core.math.Statistics | static |
| JSDist(double[] p1, double[] p2) | broad.core.math.Statistics | static |
| MAD(double[] array) | broad.core.math.Statistics | static |
| main(String[] args) | broad.core.math.Statistics | static |
| manhattanDistance(double[] x, double[] y) | broad.core.math.Statistics |  |
| max(Collection< Double > vals) | broad.core.math.Statistics | static |
| max(double[] vals) | broad.core.math.Statistics | static |
| max(int[] vals) | broad.core.math.Statistics | static |
| maxInt(Collection< Integer > vals) | broad.core.math.Statistics | static |
| maxMAD(Matrix data) | broad.core.math.Statistics | static |
| maxmean(double[] vals) | broad.core.math.Statistics | static |
| maxStdev(Matrix data) | broad.core.math.Statistics | static |
| mean(double[] values) | broad.core.math.Statistics | static |
| mean(Matrix data) | broad.core.math.Statistics | static |
| mean(Collection<?extends Number > values) | broad.core.math.Statistics | static |
| median(Collection< Double > list) | broad.core.math.Statistics | static |
| median(double[] list) | broad.core.math.Statistics | static |
| median(int[] list) | broad.core.math.Statistics | static |
| medianCollection(Collection< Double > values) | broad.core.math.Statistics | static |
| metaAvg(Collection< double[]> data) | broad.core.math.Statistics | static |
| min(Collection< Double > vals) | broad.core.math.Statistics | static |
| min(double[] vals) | broad.core.math.Statistics | static |
| min(int[] vals) | broad.core.math.Statistics | static |
| minInt(Collection< Integer > vals) | broad.core.math.Statistics | static |
| normalizeToRelativeAbundance(double[] vals) | broad.core.math.Statistics | static |
| pairedTStat(List< Double > g1, List< Double > g2) | broad.core.math.Statistics | static |
| pairedTStat(double[] gr1, double[] gr2) | broad.core.math.Statistics | static |
| pearsonCorrelation(List<?extends Number > values1, List<?extends Number > values2) | broad.core.math.Statistics | static |
| pearsonDistance(double[] x, double[] y) | broad.core.math.Statistics | static |
| pearsonDistance(List< Double > xA, List< Double > yA) | broad.core.math.Statistics | static |
| pvalue(List< Double > data, double value, boolean isSorted) | broad.core.math.Statistics | static |
| pvalue(double[] data, double value, boolean isSorted) | broad.core.math.Statistics | static |
| pvalue(List< Double > data, double value) | broad.core.math.Statistics | static |
| quantile(List<?extends Number > list, double pct) | broad.core.math.Statistics | static |
| quantile(double[] vals, double pct) | broad.core.math.Statistics | static |
| randomPermutation(int n) | broad.core.math.Statistics | static |
| rank(double[] vals, boolean ascending) | broad.core.math.Statistics | static |
| spearmanCorrelation(List< Double > xA, List< Double > yA) | broad.core.math.Statistics | static |
| spearmanCorrelation(double[] x, double[] y) | broad.core.math.Statistics | static |
| stdev(Collection<?extends Number > values) | broad.core.math.Statistics | static |
| stdev(double[] values) | broad.core.math.Statistics | static |
| sum(List< Double > vals) | broad.core.math.Statistics | static |
| sum(double[] vals) | broad.core.math.Statistics | static |
| toDoubleArray(List< Double > lst) | broad.core.math.Statistics | static |
| toDoubleArrayList(double[] intervalScores) | broad.core.math.Statistics | static |
| toUniqueSortedList(ArrayList< Double > List) | broad.core.math.Statistics | static |
| tstat(List< Double > g1, List< Double > g2, double tstat\_tuning\_param) | broad.core.math.Statistics | static |
| tstat(double[] g1, double[] g2, double tstat\_tuning\_param) | broad.core.math.Statistics | static |
| tstat(List< Double > g1, List< Double > g2) | broad.core.math.Statistics | static |
| USAGE | broad.core.math.Statistics | static |
| variance(double[] values) | broad.core.math.Statistics | static |
| variance(double[] values, double mean) | broad.core.math.Statistics | static |
| variance(List<?extends Number > values) | broad.core.math.Statistics | static |
| variance(List<?extends Number > values, double mean) | broad.core.math.Statistics | static |
| zScore(double observation, double[] controlObservations, double fudge\_factor) | broad.core.math.Statistics | static |
| zScore(double observation, double mean, double variance, int length) | broad.core.math.Statistics | static |
| zScore(double observation, double[] controlObservations) | broad.core.math.Statistics | static |
| zScore2(double observation, double[] controlObservations) | broad.core.math.Statistics | static |
| zscoreToPvalue(double zscore) | broad.core.math.Statistics | static |


---

Generated on Fri Aug 15 2014 15:10:53 for ESAT by  

 1.8.7
